# The dynamics of D-dimer level fluctuation in patients after the cemented and cementless total hip and total knee replacement

**DOI:** 10.1186/s13018-014-0089-0

**Published:** 2014-10-10

**Authors:** Piotr Bytniewski, Waldemar Machała, Leszek Romanowski, Wiesław Wiśniewski, Klaudiusz Kosowski

**Affiliations:** Department of Anaesthesiology, Health Maintenance Organization in Turek, Turek, Poland; Faculty of Physical Education and Health Preservation, The State School of Professional Higher Education in Konin, Konin, Poland; Department of Anaesthesiology and Intensive Care, Medical University of Łódź, Łódź, Poland; Department of Traumatology, Orthopaedics and Hand Surgery, Medical University of Poznań, Poznań, Poland; Department of Orthopaedics and Traumatology, Health Maintenance Organization in Turek, Turek, Poland; Health Maintenance Organization in Turek, Turek, Poland

**Keywords:** D-dimers, Total hip replacement, Total knee replacement

## Abstract

**Introduction:**

The number of total hip and total knee replacement procedures performed worldwide has tended to surge in recent years, due to the combination of such factors as the increased life expectancy, improved quality of life, advances in medical technology as well as pre-operative and post-operative patient management.

Numerous studies confirm that patients undergoing major orthopaedics procedures involving lower extremities, for instance total hip and total knee replacement, constitute the highest risk group for the development of post-operative venous thromboembolism (VTE), primarily manifested as deep vein thrombosis (DVT).

**Purpose:**

The purpose of the research was to assess the dynamics of D-dimer level fluctuation during the post-operative period in patients after the cemented or cementless total hip replacement (THR) or total knee replacement (TKR), in order to prove or reject the thesis that the cemented and cementless THR or TKR affects the post-operative D-dimer levels.

**Material and methods:**

The study group consisted of 47 patients aged 29–82 years. Of them, 23 had the cementless THR, 12 subjects had the cemented THR and another 12 patients had the TKR. All of the patients performed to measure the concentration of D-dimers in the peri-operative period at predetermined time points. For the peri-operative period was adopted from time 1 day before surgery to 10-day hospitalization. The subarachnoid block (SAB) was performed in all patients.

**Results:**

The distribution of D-dimer values throughout the entire post-operative period (up to 10th post-operative day) followed the sinusoid pattern with two peaks in all patients. It was not specific in any group.

**Conclusions:**

1. The D-dimer level almost doubles during the post-operative period in patients after THR or TKR.
2. Higher level of D-dimers in post-operative period in the research group of patients does not relate to higher risk of thromboembolic disease.

## Introduction

D-dimers are fibrin degradation products formed as a result of fibrin clot dissolution by plasmin. They participate in the entire homeostasis mechanism including coagulation and fibrinolysis processes. Under physiological conditions, both processes should take place simultaneously, maintaining some balance.

As long as the processes are normal and balanced, the body is protected against bleeding as well as against thrombus formation. When bleeding occurs, the appropriate mechanisms are activated leading to thrombus formation out of fibrin, which stops the bleeding. After the thrombus is dissolved by plasmin, the fibrin degradation products such as D-dimers are released. Their elevated plasma concentration indicates that the coagulation and fibrinolysis mechanisms were triggered. D-dimer level measurement is not included in the routine pre-operative laboratory panel. This test is typically done in patients with suspected venous thromboembolism (VTE), manifested as deep vein thrombosis (DVT) or pulmonary embolism (PE) [1]. But it may also be used as a part of diagnosis or monitoring of patients with disseminated intravascular coagulation (DIC) [2]. The term venous thromboembolism encompasses two elements: deep vein thrombosis and pulmonary embolism. It implies that while deep vein thrombosis is the primary manifestation of the disease, pulmonary embolism may follow as the sequelae.

Numerous studies confirmed that patients undergoing major orthopaedic procedures involving lower extremities, for instance total hip and total knee replacement, constitute the highest risk group for the development of post-operative venous thromboembolism, primarily manifested as the deep vein thrombosis [3-5]. The number of total hip and total knee replacement procedures performed worldwide has tended to surge in recent years, due to the combination of such factors as the increased life expectancy, improved quality of life, advances in medical technology as well as pre-operative and post-operative patient management. Although D-dimers are specific as fibrin degradation products, fibrin itself is not specific for venous thromboembolism only. The increased fibrin production, which is manifested by the elevated D-dimer concentration, can also be observed in other clinical conditions, such as cancer, infection, inflammation, surgery, injuries, haemorrhages, extensive burns, cerebral stroke, aortic aneurysm rupture, ischemic heart disease, myocardial infarction (MI), rheumatoid arthritis (RA), pregnancy and postpartum period as well as in elderly patients, patients with haematomas, extensive bruises and many others [3,6,7].

The prospective studies show that the negative result of D-dimer assay, that is the value below the cutoff point (usually 500 ng/ml), makes it possible to exclude the disease and to refrain safely from anticoagulation therapy in patients with moderate or low clinical potential for the venous thromboembolism, assessed using Wells prediction rules [8-11]. On the other hand, the elevated D-dimer level does not have a confirmatory value for venous thromboembolism due to low specificity of the assay. Therefore, the assay should not be used in order to diagnose DVT or PE, as not every patient with the elevated D-dimer level has an acute episode of VTE.

### Purpose

The purpose of the research was to assess the dynamics of D-dimer level fluctuation during the post-operative period in patients after the cemented or cementless total hip replacement (THR) or total knee replacement (TKR), in order to prove or reject the thesis that the cemented and cementless total hip or total knee replacement affects the post-operative D-dimer levels.

## Material and methods

Fifty patients (35 women and 15 men) aged 29–82 years were enrolled in the study. All subjects gave their informed consent to participate in the study (informed consent form, patient information form). Twenty-three of them had the cementless THR, 15 subjects had the cemented THR and another 12 patients had the TKR. All procedures were performed in the Department of Orthopedics and Traumatology, Health Maintenance Organization in Turek. Three patients after the cemented THR were eventually excluded from the analysis, due to the impossibility to perform the D-dimer measurements at all required time points as a result of technical difficulties. Therefore, the final study sample consisted of 47 subjects (32 women and 15 men). The study protocol was approved by the Institutional Review Board at the Karol Marcinkowski Medical University of Poznań, by the virtue of Resolution no. 739/11.

Inclusion criteria:

- Degenerative hip or knee lesions, making the patient eligible for surgery
- Category I–III at the assessment of physical status classified according to the system developed by the American Society of Anaesthesiology (ASA)
- Health insurance holders (National Health Fund)

Exclusion criteria:

- Current diagnosis or a history of VTE
- Joint injury (involving the joint scheduled for surgery), within the last few days
- Infection
- Cancer
- Pregnancy
- Use of oral anticoagulants
- ASA category > III
- Age <18 years
- Legal incapacity

Detailed medical history was obtained, and physical examination was performed in each patient prior to the scheduled surgery. The anamnesis involved the history of chronic diseases and surgery, all chronic comorbidities and their treatment as well as currently used VTE prevention. The respiratory and cardiovascular function was assessed during the physical examination. At the same time, the lower extremities were evaluated for varicose veins, and the type of total hip/knee implant was determined. Prior to the surgery, the following assays were routinely performed. None of the enrolled patients was diagnosed with venous thromboembolism based on clinical assessment and venous ultrasound scan. The perioperative period was referred to as the period from the 1st day preceding the surgery until the 10th day post-operatively, although this definition is broader than the ones commonly accepted in anaesthesiology.

In the whole peri-operative period, the D-dimer level in blood plasma was assessed by immunoenzymatic automated quantity test VIDAS D-Dimer Exclusion carried out in the immunoanalyzer VIDAS and based on marking fibrin degradation products (FbDP) in human blood plasma (with sodium citrate) with the use of enzyme-linked fluorescent assay (ELFA) technique. According to the data provided by the manufacturer (Bio-Merieux, Lyon, France), measuring range VIDAS D-Dimer Exclusion area from 45 ng/ml to 10,000 ng/ml cutoff point of 500 ng/ml, the sensitivity of about 100%, a negative predictive value (NPV) of about 100% and a specificity of about 40% [12].

The following time points have been assumed for the assays:

I. On the day preceding surgery—the pre-operative assay # “0”
II. 12 h post-operatively—assay # “1”
III. On the 1st post-operative day—assay # “2”
IV. On the 2nd post-operative day—assay # “3”
V. On each consecutive day, until the 10th post-operative day—final assay # “11”

All enrolled patients received VTE prevention, which involved administration of low molecular weight heparin at doses recommended by the Polish Society of Orthopaedics and Traumatology [13]. The preventive heparin use was prolonged up to 6 weeks post-operatively. The premedication administered approx. 40–50 min prior to the scheduled surgery involved Midazolam p.o. or i.m. at the dose of 0.07–0.1 mg/kg. Moreover, each patient received 1.0 g of Metamizole sodium dissolved in 500 ml of saline as an intravenous (i.v.) infusion. The subarachnoid block (SAB) was done in all patients using a single puncture technique with the patient sitting.

All patients were administered 1,000 mg of Tranexamic acid i.v. and infusion fluids at the dose of 20 ml/kg (i.e. approx. 1,500–2,000 ml) during the surgery. The administered infusion fluids comprised specifically of: 10% hydroxyethyl starch (HES) 200/0.5, 500 ml; the multielectrolyte solution, 500 ml; 0.9% sodium chloride (saline), 500 ml plus potentially another 500 ml bag. Moreover, if necessary, packed red blood cells, hydrocortisone (for cemented implants), sedatives (Midazolam) or other drugs were administered i.v. based on the physician’s judgement. Atropine sulphate was also administered in cases of heart rate drop by 25% as compared to baseline value. Similarly, ephedrine hydrochloride was administered in cases of mean blood pressure drop by 25% as compared to the baseline value determined during the first measurement. During the anaesthesia, the measures were taken to prevent the blood pressure drop by 35% as compared to the baseline value.

### Statistical analysis

As most of the studied variables did not follow normal distribution, the following non-parametric tests were used for the analysis:

- In order to verify the differences between the D-dimer levels at various time points within the same patient group, the Wilcoxon signed rank test was used.
- In order to verify the differences in percentage changes in D-dimer levels between the patient groups, the Mann-Whitney *U*-test was used.
- In order to assess the correlation between the baseline D-dimer level and the patient age, the Spearman’s rank correlation coefficient was calculated.

The *p* level of < .05 was assumed for all comparisons.

## Results

Characteristics for all sampled groups of patients: cementless total hip replacement (cementless THR), TKR, cemented total hip replacement (cemented THR) in terms of gender, age, BMI, past history expressed and evaluated by the international ASA scale are presented in Table 1.

**Table 1:** **Characteristics of the population of patients in terms of gender, age and including factors BMI and international ASA scale**

	**Characteristics of the population of patients**		
All patients in study (47)			
Sex (%)	Female	Male	
	68.085	31.914	
Subjects	32	15	
	ASA I	ASA II	ASA III
	12.766	31.915	55.319
	6	15	26
	Min	Max	Mean value
Age in years	29	82	66.1
BMI (kg/m2)	21.273	41.207	29.557
Cementless total hip replacement (23)			
Sex (%)	Female	Male	
	60.87	39.13	
Subjects	14	9	
ASA (%)	ASA I	ASA II	ASA III
	26.087	30.435	43.478
Subjects	6	7	10
	Min	Max	Mean value
Age in years	29	75	58.696
BMI (kg/m2)	21.273	38.587	28.953
Total knee replacement (12)			
	Female	Male	
	66.667	33.333	
Subjects	8	4	
ASA (%)	ASA I	ASA II	ASA III
	0	50	50
Subjects	0	6	6
	Min	Max	Mean value
Age in years	56	79	66.917
BMI (kg/m2)	27.349	35.467	30.967
Cemented total hip replacement (12)			
Sex (%)	Female	Male	
	83.333	16.667	
Subjects	10	2	
	ASA I	ASA II	ASA III
	0	16.667	83.333
	0	2	10
	Min	Max	Mean value
Age in years	58	82	72.667
BMI (kg/m2)	25.368	41.207	29.307

The pre-operative D-dimer levels are presented in Table 2. The baseline D-dimer level exceeded the reference value of 500 ng/mL in 37 out of 47 subjects. In most cases, this excess was attributed to patient age and their comorbidities. The D-dimer levels in all subjects, measured 12 h post-operatively are presented in Table 2.

**Table 2:** **Growth differentiation of D-dimer levels in each group of patients**

**The D-dimer levels (ng/m)**	**Cementless total hip replacement mean age 58.7 years**			**Total knee replacement mean age 66.9 years**			**Cemented total hip replacement mean age 72.7 years**		
	**Min**	**Max**	**Mean value**	**Min**	**Max**	**Mean value**	**Min**	**Max**	**Mean value**
1. The pre-operative D-dimer levels (ng/mL)	146.43	2,676.33	1,077.223	251.66	2,960.38	1,025.141	337.28	5,315.56	2,075.353
2. D-dimer levels measured 12 h post-operatively (ng/mL)	1,332.1	10,000.00	6,995.55	3,626.18	10,000.00	8,571.276	1,681.13	10,000.00	6,569.658
3. D-dimer levels measured post-operatively on day 1st (ng/mL)	887.97	10,000.00	3,152.338	1,497.00	10,000.00	6,232.967	917.85	7,882.89	3,478.125
4. The lowest D-dimer levels measured post-operatively on days 2 and 3 (ng/mL)	654.16	4,523.55	1,546.635	912.05	4,404.59	2,494.083	800.27	3,235.43	1,582.428
5. D-dimer levels measured post-operatively on days 3 and 4 (ng/mL)	1,312.47	4,523.55	2,536.357	1,441.38	4,404.59	3,331.502	1,050.91	3,921.62	2,174.206
6. The highest D-dimer levels measured post-operatively on days 4–10 (ng/mL)	1,723.36	7,862.14	3,879.205	2,360.15	8,110.22	5,032.932	2,422.00	10,000.00	4,422.438
7. D-dimer level measured post-operatively on day 10 (ng/mL)	898.39	9,601.73	3,268.066	1,752.18	5,839.16	3,721.137	2,005.96	8,019.52	4,029.627

The elevated D-dimer level at 12 h post-operatively was shown in 46 out of 47 subjects. It was the highest increase in D-dimer level observed throughout the entire follow-up period, which reached the peak of 10,000 ng/mL in almost half of the study group. Moreover, a statistically significant difference was found between the mean D-dimer levels at12 h post-operatively and the baseline values across all study groups (cementless THR, *p* <0.001; total knee replacement, *p* =0.002; cemented THR, *p* =0.006). The D-dimer levels in all subjects, measured on the1st post-operative day are presented in Table 2.

The D-dimer level decreased in all subjects on the 1st post-operative day compared to the level measured at 12 h post-operatively. However, the obtained values still exceeded the baseline levels. A statistically significant difference between the mean D-dimer level on the 1st post-operative day, compared to the measurement 12 h post-operatively was shown in all groups (cementless THR, *p* <0.001; TKR, *p* =0.003; cemented THR, *p* =0.008). Similarly, a statistically significant difference between the D-dimer level on the 1st post-operative day and its baseline values was found across all groups (cementless THR, *p* <0.001; TKR, *p* =0.002; cemented THR, *p* =0.034). The lowest D-dimer levels on the 2nd–3rd post-operative day are presented in Table 2.

The lowest D-dimer levels throughout the entire post-operative period were observed in all subjects on the 2nd–3rd post-operative day. A statistically significant difference between the mean D-dimer level on the 2nd–3rd post-operative day, compared to its value on the 1st post-operative day was shown in all patient groups (cementless THR, *p* <0.001; TKR, *p* =0.002; cemented THR, *p* =0.005). Furthermore, a statistically significant difference between the D-dimer level on the 2nd–3rd post-operative day and mean baseline value was shown in two patient groups (cementless THR, *p* <0.019; TKR, *p* =0.004).

The *p* value for the cemented THR group was 0.308; therefore, it was not considered statistically significant.

The D-dimer levels on the 3rd–4th post-operative day are presented in Table 2.

The subsequent rise of D-dimer level from the 3rd–4th post-operative day onwards was observed in all subjects. A statistically significant difference between the mean D-dimer level on the 3rd–4th post-operative day, compared to its value on the 2nd–3rd post-operative day was shown in all patient groups (cementless THR, *p* <0.001; TKR, *p* =0.005; cemented THR, *p* =0.002). The highest D-dimer levels measured post-operatively on days 4th–10th are presented in Table 2.

A statistically significant difference between the mean D-dimer levels on the 4–10th post-operative day, compared to the mean baseline values was observed in all groups (cementless THR, *p* <0.001; TKR, *p* =0.002; cemented THR, *p* =0.002). The D-dimer levels on the 10th post-operative day are presented in Table 2.

**Figure 1:**
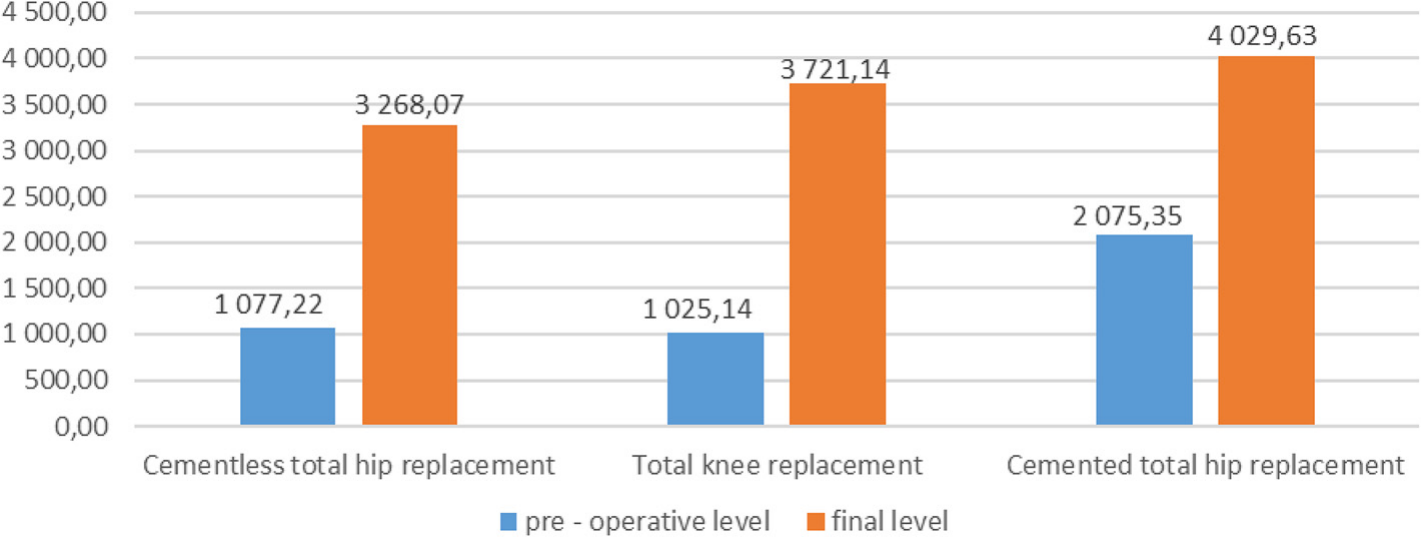
**Mean pre-operative and final (10th post-operative day) D-dimer level (ng/ml) all study groups.**

Figure 1 shows the comparison of pre-operative and final D-dimer levels. The D-dimer level on the 10th post-operative day was still almost doubled compared to the pre-operative value in most patients (Figure 1).

**Figure 2:**
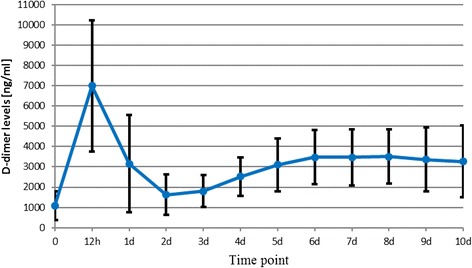
**Cementless total hip replacement.**

**Figure 3:**
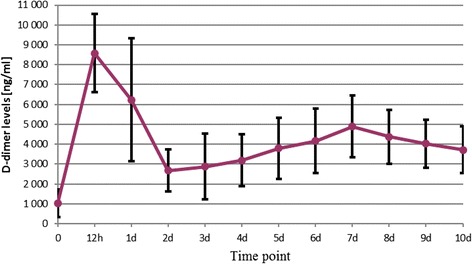
**Total knee replacement.**

**Figure 4:**
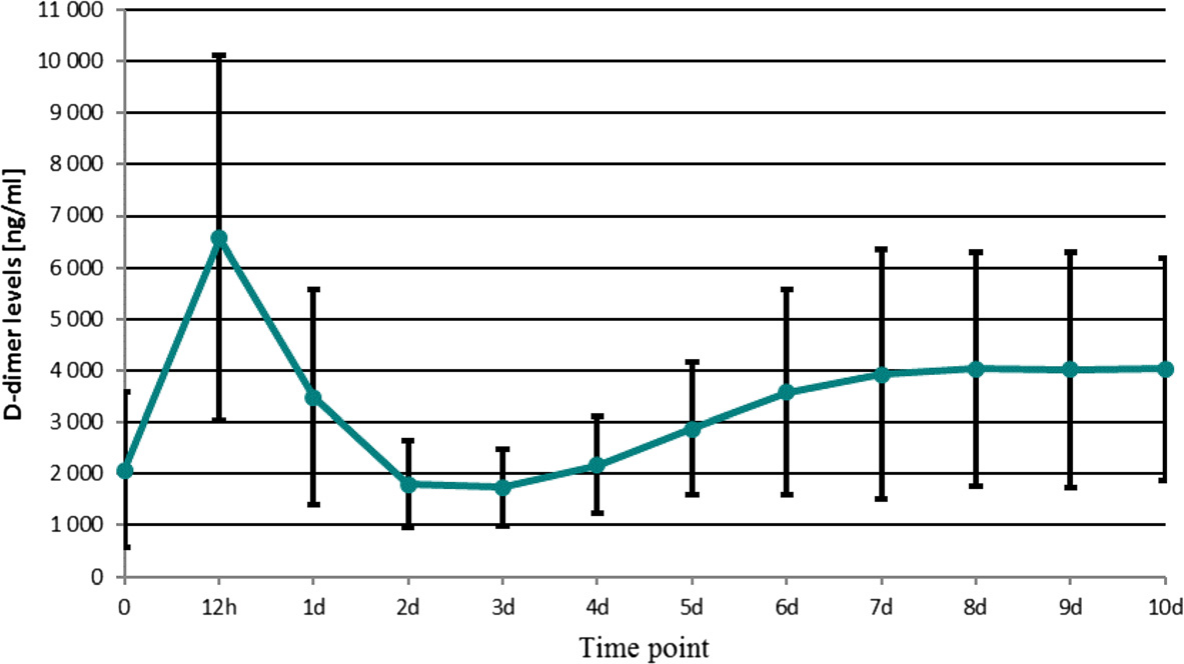
**Cemented total hip replacement.**

The D-dimer level measured post-operatively on day 10th was still almost twice as high as its pre-operative value in most patients. That is why the physicians, who continue the post-operative management of patients after THR or total knee replacement procedures, may expect the D-dimer concentrations to be significantly elevated. A statistically significant difference between the mean final D-dimer levels compared to the mean baseline (pre-operative) values was observed in all groups (cementless THR, *p* <0.001; TKR, *p* =0.002; cemented THR, *p* =0.002). Figures 2, 3 and 4 show the mean D-dimer levels for the three study groups, measured throughout the entire follow up period (the vertical bars show the mean value ± standard deviation) (Figures 2, 3 and 4).

## Discussion

Developing effective measures of venous thromboembolism detection is particularly important in patients undergoing total hip or total knee replacement, as they are at risk of contracting thrombo-embolic complications. In our research, we attempted to determine the applicability of D-dimer level measurements for the detection of venous thromboembolism in these patients. We have determined the ranges of D-dimer values measured during the post-operative period in patients after total hip or total knee replacement. These can be measured using different laboratory methods. Only these methods, characterized by the sensitivity and NPV close to 100%, considered along with the moderate or low clinical probability of VTE according to Wells prediction rule, make it possible to exclude the disease and justify the decision to refrain from treatment with anti-coagulants [8].

As the standard reference values have not been determined yet, the obtained D-dimer level results should be supplemented by the reference range for a specific type of assay. Due to the fact the reference values may be affected by multiple factors, such as patient age, comorbidities, study population or assay type, the obtained results expressed as numbers will be interpreted differently and have variable applications in different clinical settings. Moreover, D-dimer level can take a false negative value in some circumstances, e.g. at the presence of a small thrombus (in isolated distal DVT), in case of temporal delay between the onset of symptoms and the laboratory tests, or the use of anticoagulants (heparin or oral anticoagulants) [3,6,14]. The negative D-dimer values in patients with long-lasting symptoms of VTE should always be assessed with caution, as the D-dimer level may drop within 1–2 weeks following the symptom onset to only 1/4 of baseline value. However, the risk of the patient does not decrease if the disease is left untreated. Similarly, the D-dimer level decreases in response to treatment with anticoagulants; it may drop by 25% of baseline value within 24 h following the first administration of unfractionated heparin [15]. It should be emphasized that the distribution of D-dimer values throughout the entire post-operative period in our study followed the sinusoid pattern in all patients, with two peaks, and was not specific in any group.

Finally, it should be noted that the elevated D-dimer level is not enough to diagnose VTE in patients after the THR or TKR. Owing to the fact that there are no published papers discussing the issues addressed in our study, it is difficult to relate our findings to the results and observations of other authors.

## Conclusions

1. The D-dimer level almost doubles during the post-operative period in patients after THR or TKR.
2. Higher level of d-dimers in post-operative period in the research group of patients does not relate to higher risk of thromboembolic disease.

## Abbreviations

VTE: venous thromboembolism
DVT: deep vein thrombosis
DIC: disseminated intravascular coagulation
PE: pulmonary embolism
SAB: subarachnoid block
MI: myocardial infarction
RA: rheumatoid arthritis
THR: total hip replacement
TKR: total knee replacement
ASA: American Society of Anesthesiology
FbDP: fibrin degradation products
ELFA: enzyme-linked fluorescent assay
NPV: Negative predictive value
p.o.: orally
i.m.: intramuscularly
i.v.: intravenous
i.e.: that is
approx.: approximately
HES: hydroxyethyl starch
BMI: body mass index
P: level of significance
1st: first
2nd: second
3rd: third
4th: fourth
10th: tenth
